# Overview and Methods for the Youth Risk Behavior Surveillance System — United States, 2019

**DOI:** 10.15585/mmwr.su6901a1

**Published:** 2020-08-21

**Authors:** J. Michael Underwood, Nancy Brener, Jemekia Thornton, William A. Harris, Leah N. Bryan, Shari L. Shanklin, Nicholas Deputy, Alice M. Roberts, Barbara Queen, David Chyen, Lisa Whittle, Connie Lim, Yoshimi Yamakawa, Michelle Leon-Nguyen, Greta Kilmer, Jennifer Smith-Grant, Zewditu Demissie, Sherry Everett Jones, Heather Clayton, Patricia Dittus

**Affiliations:** ^1^Division of Adolescent and School Health, National Center for HIV/AIDS, Viral Hepatitis, STD, and TB Prevention, CDC; ^2^Epidemic Intelligence Service; ^3^ICF International, Rockville, Maryland; ^4^Westat, Rockville, Maryland

## Abstract

Health risk behaviors practiced during adolescence often persist into adulthood and contribute to the leading causes of morbidity and mortality in the United States. Youth health behavior data at the national, state, territorial, tribal, and local levels help monitor the effectiveness of public health interventions designed to promote adolescent health. The Youth Risk Behavior Surveillance System (YRBSS) is the largest public health surveillance system in the United States, monitoring a broad range of health-related behaviors among high school students. YRBSS includes a nationally representative Youth Risk Behavior Survey (YRBS) and separate state, local school district, territorial, and tribal school–based YRBSs. This overview report describes the surveillance system and the 2019 survey methodology, including sampling, data collection procedures, response rates, data processing, weighting, and analyses presented in this *MMWR* *Supplement*. A 2019 YRBS participation map, survey response rates, and student demographic characteristics are included. In 2019, a total of 78 YRBSs were administered to high school student populations across the United States (national and 44 states, 28 local school districts, three territories, and two tribal governments), the greatest number of participating sites with representative data since the surveillance system was established in 1991. The nine reports in this *MMWR Supplement* are based on national YRBS data collected during August 2018–June 2019. A full description of 2019 YRBS results and downloadable data are available (https://www.cdc.gov/healthyyouth/data/yrbs/index.htm).

Efforts to improve YRBSS and related data are ongoing and include updating reliability testing for the national questionnaire, transitioning to electronic survey administration (e.g., pilot testing for a tablet platform), and exploring innovative analytic methods to stratify data by school-level socioeconomic status and geographic location. Stakeholders and public health practitioners can use YRBS data (comparable across national, state, tribal, territorial, and local jurisdictions) to estimate the prevalence of health-related behaviors among different student groups, identify student risk behaviors, monitor health behavior trends, guide public health interventions, and track progress toward national health objectives.

## Introduction

Adolescence is typically a healthy period of life, and CDC reports that youths continue to make better decisions for their health (*1*). However, some high school–aged youths experience disparate health risks that increase the possibility of acquiring a sexually transmitted disease (STD), including human immunodeficiency virus (HIV) infection, and increase opportunities for substance use, mental health problems, and interpersonal violence or self-harm. Risky health behaviors practiced during adolescence often persist into adulthood (*2*). In 2018, CDC reported that the leading causes of death among U.S. adolescents were attributable to motor-vehicle crashes, followed by suicide and homicide (*3*). In contrast, that same year, a separate study reported the leading causes of death among persons of all ages were heart disease, followed by cancer and unintentional injuries (e.g., burns, drowning, falls, poisoning, and motor-vehicle crashes) (*4*).

The Youth Risk Behavior Surveillance System (YRBSS) monitors health behaviors, conditions, and experiences among high school students throughout the United States. The system includes a national Youth Risk Behavior Survey (YRBS), conducted by CDC, and separate state, local school district, territorial, and tribal school–based YRBSs, which are referred to as site-level surveys. YRBSS is designed to monitor priority health risk behaviors that contribute to the leading causes of mortality, morbidity, and social problems among youths and adults. The following categories of behaviors are included in the system: 1) behaviors that contribute to unintentional injury and violence; 2) tobacco use; 3) alcohol and other drug use; 4) sexual behaviors that contribute to unintended pregnancy and STD/HIV infection; 5) dietary behaviors; and 6) physical inactivity.

This report describes the 2019 YRBS methodology, including sampling, data collection, processing, weighting, and analyses. Results include a 2019 YRBS participation map, survey response rates (1991–2019), and student demographic characteristics from the national survey. Furthermore, this overview report is one of nine featured in this *MMWR Supplement*. Each report uses YRBS data to assess a priority public health topic among adolescents. In addition to this overview report, this supplement includes national YRBS updates regarding condom and contraceptive use; violence victimization and suicide ideation by sexual identity; interpersonal violence victimization; opioid, alcohol, and other substance use behaviors; suicide ideation and behaviors; tobacco use, including vaping; dietary behaviors and physical activity; and transportation risk behaviors. Each report might not include all national YRBS data related to the topics that were collected in 2019, and this supplement does not include any data from site-level surveys; however, all the data are publicly available. (YRBS data and documentation are available at https://www.cdc.gov/healthyyouth/data/yrbs/data.htm.) Stakeholders and public health practitioners can use YRBS data (comparable across national, state, tribal, and local jurisdictions) to estimate the prevalence of health-related behaviors among different student groups, identify student risk behaviors, monitor health behavior trends, guide public health interventions, and track progress toward national health objectives.

## National YRBS Methodology

### Overview

The national YRBS is conducted biennially during the spring of odd-numbered years and allows CDC to assess how risk behaviors change temporally among the U.S. high school population. The national YRBS provides comparable data across years and allows state and local entities conducting their own YRBS to demonstrate how the behaviors of their youths compare with those at the national level. YRBS is conducted among students in grades 9–12 who attend U.S. public and private schools. A nationally representative sample of schools and a random sample of classes within those schools are selected to participate. The survey is self-administered anonymously by using a computer-scannable questionnaire booklet and takes one class period (approximately 45 minutes) to complete.

### Questionnaire

In 2019, the YRBS questionnaire consisted of 99 questions. Eighty-nine of those questions were included in the standard questionnaire* used by sites. Ten additional questions were added to the standard questionnaire that reflect areas of interest for CDC and other stakeholders, forming the 99-question national YRBS questionnaire. As in all cycles, both the standard questionnaire and additional national-only questions were revised to ensure that emerging and prevailing risk behaviors among high school students were measured. Subject matter experts from CDC and elsewhere proposed changes, additions, and deletions to the questionnaire. New and revised questions were reviewed for format, readability, and clarity and were subjected to cognitive testing. CDC made further refinements to the questions on the basis of those testing results.

All questions, except those assessing height, weight, and race, were multiple choice, with a maximum of eight mutually exclusive response options and only one possible answer per question. The survey questions have undergone test-retest analysis and demonstrated good reliability (*5*,*6*). The wording of each question, including recall periods and response options, and operational definitions for each variable, are available by reviewing the 2019 YRBS questionnaire and data user’s guide. (YRBSS data and documentation are available at https://www.cdc.gov/healthyyouth/data/yrbs/data.htm .)

### Sampling

The 2019 YRBS sampling frame consisted of all regular public (including charter schools), parochial, and other nonpublic schools with students in at least one of grades 9–12 in the 50 U.S. states and the District of Columbia. Alternative schools, special education schools, schools operated by the U.S. Department of Defense, the Bureau of Indian Education, and vocational schools serving only students who also attended another school were excluded. Schools with an enrollment of ≤40 students across grades 9–12 also were excluded. The sampling frame was based on data sets obtained from Market Data Retrieval, Inc., and the National Center for Education Statistics (NCES). NCES data sets were based on the Common Core of Data (https://nces.ed.gov/ccd) for public schools and the Private School Universe Survey (https://nces.ed.gov/surveys/pss) for nonpublic schools.

A three-stage cluster sampling design was used to produce a nationally representative sample of students in grades 9–12 who attend public and private schools. The first-stage sampling frame comprised 1,257 primary sampling units (PSUs), consisting of entire counties, groups of smaller adjacent counties, or parts of larger counties. The 1,257 PSUs were categorized into 16 strata according to their metropolitan statistical area status (e.g., urban or rural) and the percentages of non-Hispanic black (black) and Hispanic students in each PSU. From the 1,257 PSUs, 54 were sampled with probability proportional to overall school enrollment size for that PSU. For the second-stage sampling, secondary sampling units (SSUs) were defined as a physical school with grades 9–12 or a school created by combining nearby schools to provide all four grades. From the 54 PSUs, 162 SSUs were sampled with probability proportional to school enrollment size. To provide adequate coverage of students in small schools, an additional 15 small SSUs were selected from a subsample of 15 PSUs from the 54 PSU sample. These 177 SSUs corresponded to 184 physical schools. The third stage of sampling comprised random sampling of one or two classrooms in each of grades 9–12 from either a required subject (e.g., English or social studies) or a required period (e.g., homeroom or second period). All students in sampled classes were eligible to participate. Schools, classes, and students who refused to participate were not replaced in the sampling design.

### Data Collection Procedures

CDC’s Institutional Review Board approved the protocol for the YRBS. Survey procedures were designed to protect students’ privacy by allowing for anonymous and voluntary participation. Before survey administration, local parental permission procedures were followed. During survey administration, students completed the self-administered questionnaire during one class period and recorded their responses directly on a computer-scannable booklet.

### Response Rates and Data Processing

For the 2019 YRBS, 13,872 questionnaires were completed in 136 schools. The national data set was cleaned and edited for inconsistencies. Missing data were not statistically imputed. A questionnaire failed quality control when <20 responses remained after editing or when it contained the same answer to ≥15 consecutive questions. Among the 13,872 completed questionnaires, 195 failed quality control and were excluded from analysis, resulting in 13,677 usable questionnaires. The school response rate was 75.1%; the student response rate was 80.3%; and the overall response rate (i.e., [student response rate] × [school response rate]) was 60.3%.

Race/ethnicity was ascertained from two questions: 1) “Are you Hispanic or Latino?” (with response options of “yes” or “no”) and 2) “What is your race?” (with response options of “American Indian or Alaska Native,” “Asian,” “black or African American,” “Native Hawaiian or other Pacific Islander,” or “white”). For the second question, students could select more than one response option. For this report, students were classified as Hispanic/Latino and are referred to as Hispanic if they answered “yes” to the first question, regardless of how they answered the second question. Students who answered “no” to the first question and selected only black or African American to the second question were classified as black or African American and are referred to as black. Students who answered “no” to the first question and selected only white to the second question were classified and are referred to as white. Race/ethnicity was classified as missing for students who did not answer the first question and for students who answered “no” to the first question but did not answer the second question.

To obtain a sufficient sample size for analyses of health-related behaviors by sexual identity and sex of sexual contacts, students were divided into groups (Table 1). Students who had no sexual contact were excluded from analyses related to sexual behaviors, female students who had sexual contact with only females were excluded from analyses on condom use and birth control use, and male students who had sexual contact with only males were excluded from analyses on birth control use.

**TABLE 1:** Processing of sexual identity and sex of sexual contacts questions — Youth Risk Behavior Survey, United States, 2019

Question	Student response		Analytic description
**Sexual identity**			
**Which of the following best describes you?** 1) Heterosexual (straight), 2) gay or lesbian, 3) bisexual, or 4) not sure	Heterosexual (straight) Gay or lesbian or bisexual Not sure		Heterosexual students Lesbian, gay, or bisexual students Not-sure students
**Sex of sexual contacts**			
**During your life, with whom have you had sexual contact?** 1) I have never had sexual contact, 2) females, 3) males, or 4) females and males **What is your sex?** 1) Male or 2) female	I have never had sexual contact*		Students who had no sexual contact
	**Contact:** Female Male	**Student:** Male Female	Students who had sexual contact with only the opposite sex
	**Contact:** Male Females and males Female Females and males	**Student:** Male^†^ Male Female^†^ Female	Students who had sexual contact with only the same sex or with both sexes

* Excluded from analyses on sexual behaviors.

^†^ Excluded from analyses on birth control use and condom use.

### Weighting

A weight based on student sex, race/ethnicity, and grade was applied to each record to adjust for school and student nonresponse and oversampling of black and Hispanic students. The overall weights were scaled so that the weighted count of students equals the total sample size, and the weighted proportions of students in each grade match the national population proportions. Therefore, weighted estimates are nationally representative of all students in grades 9–12 attending U.S. public and private schools.

### Analytic Methods

Findings presented in this *MMWR Supplement* and Youth Online (https://nccd.cdc.gov/Youthonline/App/Default.aspx), an interactive data analysis tool that allows access to all YRBSS data, follow analytic methods similar to what is described in this overview report. For more information regarding the analyses presented in this supplement (e.g., variables analyzed, custom measures, and data years), see the Methods section in each individual report.

All statistical analyses were conducted on weighted data by using SAS (version 9.4; SAS Institute) and SUDAAN (version 11.0.1; RTI International) software to account for the complex sampling designs. In all reports, prevalence estimates and confidence intervals were computed for variables in the YRBS data set. Pairwise differences between populations (e.g., sex, race/ethnicity, grade, sexual identity, and sex of sexual contacts) were determined using *t-*tests. Prevalence estimates were considered statistically significant if the *t-*test p value was <0.05.

In reports that analyzed data related to temporal trends, prevalence estimates for variables assessed with identically worded questions were examined. Logistic regression analyses were used to account for all available estimates; control for sex, grade, and racial/ethnic changes over time; and assess long-term linear and quadratic trends. A p value associated with the regression coefficient that was <0.05 was considered statistically significant. Linear and quadratic time variables were treated as continuous and were coded by using orthogonal coefficients calculated with PROC IML in SAS. A minimum of 3 survey years was required for calculating linear trends, and a minimum of 6 survey years was required to calculate quadratic trends. Separate regression models were used to assess linear and quadratic trends for every variable. When a significant quadratic trend was identified, Joinpoint software was used to automate identification of the year when the nonlinear (i.e., quadratic) trend changed. Regression models were used to identify linear trends occurring in each segment. Cubic and higher-order trends were not assessed. A quadratic trend indicates a statistically significant but nonlinear trend in prevalence over time. A long-term temporal change that includes a significant linear and quadratic trend demonstrates nonlinear variation (e.g., leveling off or change in direction) in addition to an overall increase or decrease over time.

In reports that analyzed 2-year changes in health-related behaviors, prevalence estimates from 2017 and 2019 were compared by using *t*-tests for variables assessed with identically worded questions in both survey years. Prevalence estimates were considered statistically different if the *t*-test p value was <0.05.

### Data Availability and Dissemination

YRBS data (1991–2019) are available from the YRBSS data and documentation website (https://www.cdc.gov/healthyyouth/data/yrbs/data.htm), as are additional resources, including data documentation and analysis guides. Data are available in both Access and ASCII formats. SAS and SPSS programs are provided for converting the ASCII data into SAS and SPSS data sets. Variables are standardized to facilitate trend analyses and for combining data. YRBSS data are also available online by using Youth Online (https://nccd.cdc.gov/Youthonline/App/Default.aspx), a tool that allows point-and-click data analysis and creation of customized tables, graphs, maps, and fact sheets. Youth Online also performs statistical tests by health topic and filters and sorts data by race/ethnicity, sex, grade, and sexual orientation (sexual identity and sex of sexual contacts). Finally, YRBS Explorer is a new application featuring user-friendly options to view and compare national, state, and local data via tables and graphs (https://yrbs-explorer.services.cdc.gov). Data requests and other YRBSS-related questions can be sent to CDC by using the data request form (https://www.cdc.gov/healthyyouth/data/yrbs/contact.htm).

## State, Local School District, Territorial, and Tribal YRBS Methodology

### Overview

Biennial administration of site-level YRBSs allows state and local education and health agencies to assess how risk behaviors change temporally among the high school population in their respective jurisdiction. Site-level YRBS data provide comparable data across years and allow comparisons of student behaviors across jurisdictions (e.g., national or state). Site-level surveys are conducted among students in grades 9–12 attending public schools by using samples representative of the state, local, territorial, or tribal jurisdiction where they are administered. The survey is self-administered anonymously and takes one class period (approximately 45 minutes) to complete. State and local institutional review boards approved the protocol for their respective YRBSs. Survey methodology for data collection, processing, and analytic methods were the same as those described for the national YRBS.

### Questionnaires

The 2019 YRBS standard questionnaire contained 89 questions and was used as the starting point for site-level YRBS questionnaires. Sites could add or delete questions but were required to use at least 60 of the questions on the standard questionnaire. This flexibility allows YRBS coordinators and other local stakeholders the opportunity to pursue topics of interest by customizing their survey.

### Sampling

Sites used a two-stage cluster sampling design to produce a representative sample of students in grades 9–12 in their jurisdiction. In 41 states, three local school districts, and one territory, in the first sampling stage, public schools with any of grades 9–12 were sampled with probability proportional to school enrollment size. In two states, 25 local school districts, and two territories, all schools in the jurisdiction were selected to participate (i.e., a census of schools). In the second sampling stage, intact classes from either a required subject (e.g., English or social studies) or a required period (e.g., homeroom or second period) were sampled randomly. In three sites (Vermont, the District of Columbia, and Palau), a census of students was eligible to participate.

### Response Rates and Nonresponse Bias Analyses

Before the 2019 YRBS cycle, CDC required a minimum 60% overall response rate for data from a jurisdiction to be weighted. As response rates in federal surveys continue to decline (*7*), a better understanding of the complex association between nonresponse and nonresponse bias is needed. In 2019, CDC chose three YRBS sites with overall response rates of 50%–60% (Nebraska; Texas; and Spartanburg County, South Carolina) to pilot nonresponse bias analyses to evaluate data representativeness. Because of data limitations, comparisons were limited to responding and nonresponding schools by school size and responding and nonresponding students by grade. Weighted sample and population percentages by grade, sex, and race/ethnicity were also compared. Overall, few statistically significant differences between comparison groups were found, which suggested that the data were generally representative of their respective populations. For the 2019 cycle, CDC used nonresponse bias analysis results to help determine whether data were weighted for sites with overall response rates <60%.

### Weighting

YRBS data were weighted if sites collected data from a representative sample of students (determined either by an overall response rate of ≥60% or nonresponse bias analysis indicating no significant bias). A weight based on student sex, race/ethnicity, and grade was applied to each record to adjust for school and student nonresponse in each jurisdiction. The weighted count of students equals the student population in each jurisdiction. Data from 44 states and 28 local school districts were weighted. In 26 states and 13 local school districts, weighted estimates are representative of all students in grades 9–12 attending regular public schools, and in 13 states and eight local school districts, weighted estimates are representative of regular public school students plus students in grades 9–12 in other types of public schools (e.g., alternative or vocational schools).

### Data Availability and Dissemination

A combined data set including national, state, local school district, territorial, and tribal YRBS data (1991–2019) is available from the YRBSS data and documentation website (https://nccd.cdc.gov/Youthonline/App/Default.aspx). Availability of site data depends on survey participation, data quality, and data-sharing policies. Information about YRBSS data is available at the participation maps and history website (https://www.cdc.gov/healthyyouth/data/yrbs/participation.htm). Data requests and other YRBS-related questions can be sent to CDC by using the data request form. (The YRBSS question, comment, and data request form is available at https://www.cdc.gov/healthyyouth/data/yrbs/contact.htm.) Site-level YRBS data (from high school and middle school surveys) collected during 1991–2019 are available through Youth Online (https://nccd.cdc.gov/Youthonline/App/Default.aspx) and YRBS Explorer (https://yrbs-explorer.services.cdc.gov).

## YRBS Response Rates and 2019 Demographic Characteristics

**FIGURE 1:**
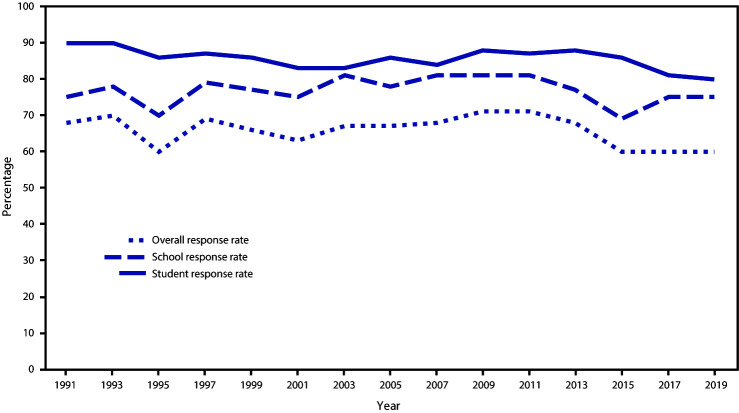
Overall, school, and student response rates for the national Youth Risk Behavior Surveys — United States, 1991–2019

During 1991–2019, national YRBS overall response rates remained at >60% (Figure 1). They reached a high of 71% during the 2009 and 2011 YRBS cycles, followed by steady decreases; response rates have remained in the low 60% range during the 2015–2019 cycles. Since 1991, school response rates have varied from 70% to the low 80% range, whereas student participation rates have been consistent at 80%–90%. 

Data were weighted to match national population proportions. Thus, 50.6% of students were male, and 26.6% were in 9th grade; 25.5% were in 10th grade; 24.2% were in 11th grade; and 23.5% were in 12th grade (Table 2). In regard to race/ethnicity, the majority of students were no-Hispanic white (white) (51.2%), followed by Hispanic (26.1%), black (12.2%), and other (10.6%), which is defined as American Indian or Alaska Native, Asian, Native Hawaiian or other Pacific Islander, or multiracial but non-Hispanic.

**TABLE 2:** Youth Risk Behavior Survey student demographic characteristics — United States, 2019

Characteristic	No. (%)
**Participating schools**	**136 (100)**
**Student sample size**	13,677* (100)
**Response rates**	
Schools	(75.1)
Students	(80.3)
Total	(60.3)
**Sex^†^**	
Male	6,641 (50.6)
Female	6,885 (49.4)
**Race/Ethnicity^†,§^**	
White, non-Hispanic	6,668 (51.2)
Black, non-Hispanic	2,040 (12.2)
Hispanic	3,038 (26.1)
Other	1,493 (10.6)
**Grade^†,§^**	
9	3,637 (26.6)
10	3,717 (25.5)
11	3,322 (24.2)
12	2,850 (23.5)

* Among the 13,872 completed questionnaires, 195 failed quality control and were excluded from analysis, resulting in 13,677 usable questionnaires.

^†^ Does not included students who responded “ungraded” or “other grade.”

^§^ Percentages might not total 100% because of rounding.

Nationwide, 84.4% of students self-identified as heterosexual, 2.5% as gay or lesbian, and 8.7% as bisexual; 4.5% were not sure of their sexual identity (Table 3). In 2019, 45.4% of students had sexual contact with only the opposite sex, 2.2% with only the same sex, and 4.8% with both sexes; 47.6% had had no sexual contact.

**TABLE 3:** Number and percentage of students, by sexual identity and sex of sexual contacts — Youth Risk Behavior Survey, United States, 2019

Characteristic	Total		Male		Female	
	No. (%)	95% CI	No. (%)	95% CI	No. (%)	95% CI
**Sexual identity**						
Heterosexual	**10,853 (84.4)**	**83.4–85.3**	5,728 (91.2)	90.1–92.3	5,048 (77.6)	75.9–79.3
Gay or lesbian	**380 (2.5)**	**2.1–3.0**	157 (2.1)	1.6–2.7	211 (2.9)	2.3–3.6
Bisexual	**1,151 (8.7)**	**8.0–9.4**	201 (3.4)	2.8–4.1	929 (13.9)	12.7–15.2
Not sure	**591 (4.5)**	**3.9–5.0**	223 (3.2)	2.7–3.9	350 (5.6)	4.7–6.6
**Sex of sexual contacts**						
Opposite sex only	**4,856 (45.4)**	**42.8–48.1**	2,642 (49.5)	46.2–52.8	2,214 (41.3)	38.7–44.0
Same sex only	**292 (2.2)**	**1.8–2.7**	99 (1.6)	1.2–2.0	193 (2.8)	2.2–3.6
Both sexes	**526 (4.8)**	**4.2–5.5**	90 (1.8)	1.4–2.3	436 (7.8)	6.7–9.1
No sexual contact	**4,953 (47.6)**	**44.8–50.4**	2,346 (47.1)	43.9–50.4	2,607 (48.0)	45.1–50.9

**Abbreviation:** CI = confidence interval.

### 2019 Site-Level YRBS Participation and Student Response Rates

**FIGURE 2:**
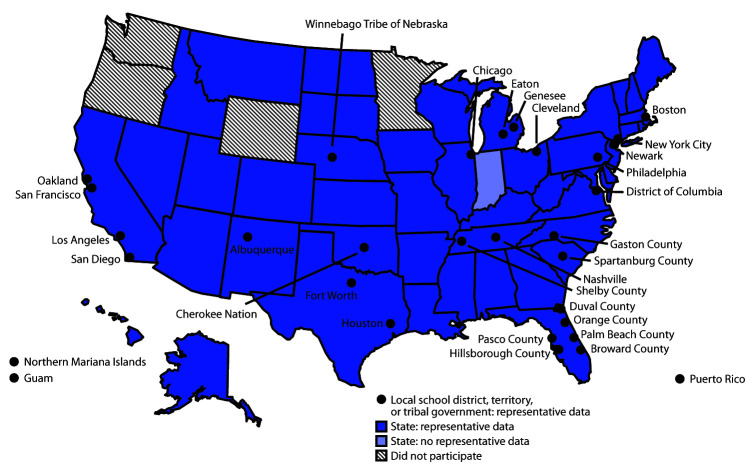
State, local school district, territorial, and tribal government Youth Risk Behavior Surveys — selected U.S. sites, 2019

**FIGURE 3:**
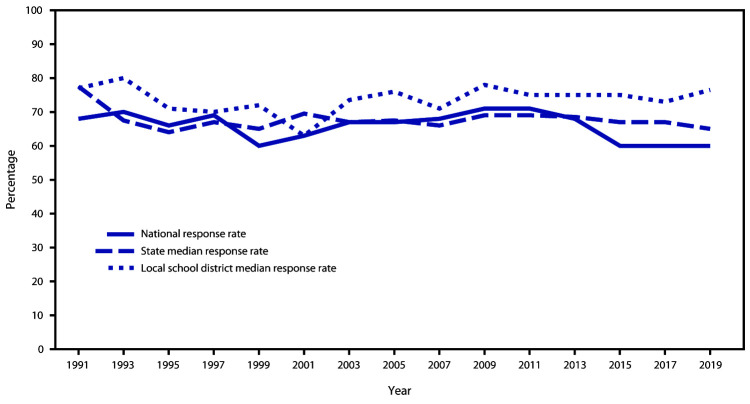
National, state, and local school district Youth Risk Behavior Survey response rates — United States and selected U.S. sites, 1991–2019* * Does not include Youth Risk Behavior Survey data from U.S. territories and tribal governments.

**FIGURE 4:**
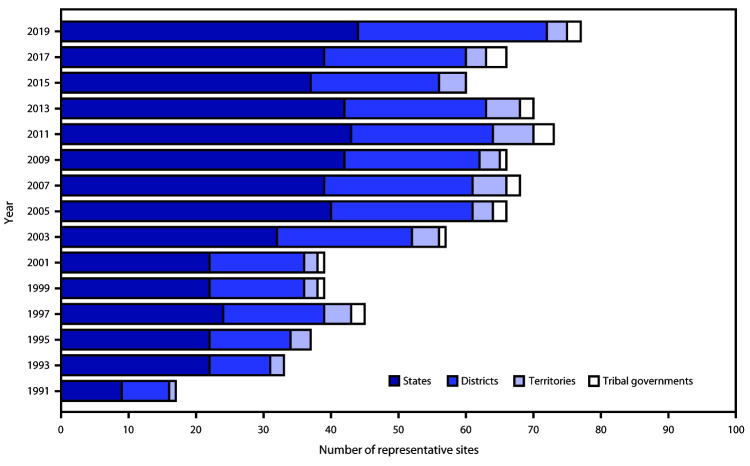
Number of states, local school districts, territories, and tribal governments with representative Youth Risk Behavior Survey data, by year of survey — selected U.S. sites, 1991–2019

In 2019, a total of 44 states, 28 local school districts, three territories, and two tribal governments had representative data (Figure 2). In 2019, the median response rate for state YRBSs with representative data was 65.0% (Figure 3), which has typically remained at 60%–70% since 1991. The median response rate for local school district YRBSs with representative data was 76.5% (Figure 3) and has typically remained at 70%–80% since 1991. Since the inception of YRBSS in 1991, the number of sites with representative data has increased, reaching a high of 77 in 2019 (Figure 4).

## Discussion

YRBSS is the largest public health surveillance system in the United States, monitoring multiple health-related behaviors among high school students. Since 1991, YRBSS has collected data from approximately 4.9 million high school students in approximately 2,100 separate surveys. Survey response rates have remained slightly above 60%, since YRBSS inception. Consistent and relatively high response rates allow for long-term trend analyses of student health behaviors and experiences. During the 2019 cycle, 78 separate jurisdictions successfully collected YRBS data from a broad diversity of high school students. Nationally representative data from adolescents of various demographic profiles (e.g., sex, race and ethnicity, sexual identity) provide information regarding disparities in health-related behaviors and highlight long-term trends in the prevalence of these behaviors. 

In 2019, CDC launched the Public Health Data Modernization Initiative to enhance the potential of using data for disease detection and elimination. The initiative envisions a future in which data drives action efficiently, flexibly, rapidly, and with impact. CDC leverages technology, knowledge, leadership, access, and collaboration to harness the life-saving power of data. YRBSS has both longstanding and newly implemented features that align with the modernization initiative. CDC scientists provide technical support to help state and local education and health agencies administer their YRBS. Flexibility in the questionnaire design process allows stakeholders to collect data of interest across student populations. Detailed YRBS site reports are rapidly returned to state and local departments of health and education, often within 16 weeks of survey administration. In 2019, YRBSS reach (measured by the number of sites with representative data) has increased to 78 sites including the national survey, the most in YRBSS history. These data will help identify student risk behaviors, affect decision-making, and guide public health interventions.

The public release of YRBS data coincides with the publication of this nine-part *MMWR* *Supplement* and is an agencywide collaboration. Subject matter experts from selected CDC programs contributed to this supplement to highlight public health concerns among U.S. high school students. YRBS data dissemination is managed through online requests, Youth Online, and YRBS Explorer. This year, CDC updated Youth Online to strengthen data presentation, improve user experience, and ultimately expand reach for YRBS data. These improvements to data dissemination will improve YRBS access, expand usage, and maximize impact.

CDC continually works to strengthen YRBSS, and new developments are under way. In 2019, CDC launched a project to update reliability testing for the national YRBS questionnaire. As other school-based surveys move toward electronic platforms (e.g., computer, smart phone, or tablet), some site-level YRBSs have also transitioned to electronic survey administration. CDC recently completed pilot testing for a tablet-based survey administration of the YRBS questionnaire and is considering using tablets for future YRBSs. Finally, CDC is exploring innovative analytic methods to stratify YRBS data by school-level socioeconomic status and geographic location. A recent study using this approach reported students attending schools in low socioeconomic areas were more likely to experience violence, poor emotional well-being, and suicidality (*8*).

## Limitations

Reports in this supplement include a limitations section describing the analyses pertaining to that particular report. In general, YRBSS findings are subject to at least six limitations. First, these data apply only to youths who attend school and therefore are not representative of all persons in this age group. In 2019, approximately 5% of high school–aged youths (ages 14–17 years) were not enrolled in school (*9*). Those youths might engage in riskier health behaviors than their peers, and those behaviors are not captured in the school-administered YRBS. Second, the extent of underreporting or overreporting of health-related behaviors cannot be determined, although the survey questions demonstrate good test–retest reliability (*5*,*6*). Third, not all states and local school districts administer YRBS, and those that did administer it might not include all the standard questions on their YRBS questionnaire; therefore, data for certain variables are not available for some sites. Fourth, YRBS data analyses are based on cross-sectional surveys and can only provide an indication of association, not causality. Moreover, the survey is descriptive and not designed to explain the reasons behind any observed trends. Fifth, limitations exist related to assessment of sexual and gender identity. Students might not be fully aware of their sexual identity at the time of assessment or might not have understood the sexual identity question. The category of students who are not sure of their sexual identity might encompass students who are unsure of their sexuality, students who were uncomfortable answering the question, or students who did not understand the question. In addition, although some sites asked questions about transgender students, the national YRBS does not include a question about gender identity; therefore, national prevalence estimates for this population of students cannot be assessed. Finally, a limitation exists regarding the aggregation of race and ethnicity data. The national YRBS aggregates these data into broad categories of white, black, and Hispanic. All other students are classified as “other.” More detailed racial/ethnic information, as published elsewhere, provides valuable data regarding health disparities among high school students (*10*). 

## Conclusion

YRBSS is the best source for quality data at the national, state, territorial, tribal, and local school district levels for monitoring health-related behaviors that contribute to the leading causes of mortality and morbidity among U.S. high school students and that can lead to health problems as adults. A recent report from the National Academies of Sciences, Engineering, and Medicine used YRBS as its data source on the basis of the strengths of the system (*11*). In 2019, in addition to the national data, 44 states, 28 local school districts, three territories, and two tribal governments received data representative of their high school student populations.

This overview report describes YRBSS methods for guiding the analyses presented in this *MMWR* *Supplement*. A full description of 2019 YRBS results and downloadable data are available (https://www.cdc.gov/healthyyouth/data/yrbs/index.htm).

## Footnotes

* The standard YRBSS questionnaire includes 89 questions. YRBS coordinators (located in CDC-funded states, local school districts, territories, and tribes) voted for or against each proposed change, addition, and deletion. Final content of the standard YRBS questionnaire was decided on the basis of the results of this voting process.
